# Sugar content and erosive potential of commonly prescribed Orodispersible tablets- An *in vitro* study.

**DOI:** 10.12688/f1000research.130786.2

**Published:** 2023-04-12

**Authors:** Lahari Anand, Kalyana Pentapati, Revathi Shenoy, Geethika Yelleti, Saurabh Kumar

**Affiliations:** 1 Public Health Dentistry, Manipal College of Dental Sciences, Manipal Academy of Higher Education, Manipal Academy of Higher Education, Manipal, Karnataka, 576104, India; 2 Biochemistry, Kasturba Medical College, Manipal, Manipal Academy of Higher Education, Manipal, Karnataka, 576104, India; 3 Pedodontics and Preventive Dentistry, Manipal College of Dental Sciences, Manipal, Manipal Academy of Higher Education, Manipal, Karnataka, 576104, India

**Keywords:** Oral, Dispersible tablets, pH, Sugar content, Erosive potential

## Abstract

**Background: **Dental caries is a major non-communicable disease of public health concern caused due to freely available dietary sugars. We aimed to compare the sugar content and erosive potential with duration of use and drug classes of orodispersible tablets (ODTs).

**Methods: **We conducted an *in vitro* evaluation of the total sugar content (TSC), Potential of Hydrogen (pH), solubility, and Titratable Acidity (TA) of commonly prescribed 62 ODTs. TA was measured by titrating the samples with known amount of. 0.1N sodium hydroxide (NaOH) with phenolphthalein indicator and pH was determined by digital pH meter. TSC was evaluated by phenol sulphuric acid. Solubility was assessed by filtration.

**Results: **Out of the 62 ODTs, majority were Antimicrobials (n=30). One-quarter of the ODTs (26%) had a mean pH below ≤5.5. No significant difference was seen in the mean pH with respect to different drug classes (p=0.082) and duration of use of ODTs. A significant difference was seen in the mean percentage solubility with respect to drug classes (p<0.001). Antimicrobials had the least percentage of solubility as compared to other drug classes. Antiemetics and proton pump inhibitors (24.33 ± 17.34) had significantly higher mean percentage sugar content than Antimicrobials (23.25 ± 17.16). No significant difference was seen in the mean TSC with respect to various drug classes (p=0.718) and between the duration of use of drugs (P=0.568) respectively. No significant difference was seen in the mean percentage TA with respect to drug class (p=0.123) and duration of use of drugs (p=0.424).
** **

**Conclusion: **Overall, we can conclude that one in four ODT formulations had a pH below 5.5 (critical pH).  Only one ODT formulation did not have a sugar content. No difference was seen in the mean pH, sugar content, and TA with respect to duration of use of drugs and drug classes.

## Introduction

Dental caries is a major and widespread non-communicable disease of public health concern. ^1^ Freely available sugars in a person’s diet are a major risk factor in the etiology of caries which has a detrimental effect throughout the life (more information is available from the World Health Organisation (WHO) ( https://www.who.int/news-room/fact-sheets/detail/sugars-and-dental-caries). Children have less immunity, thus making them vulnerable to chronic diseases which requires administration of medicines. Children who are unwell for an extended period may be more prone for developing caries, and more dietary sugars may increase the likelihood and severity of it. ^2^

Many liquid oral medications (LOM) contain readily available sugars like sucrose and glucose. ^3^ The requirement for easily adjustable dosage forms is one of the most frequent issues in pediatric pharmacology. ^4^ This issue has long been resolved by the development and use of liquid preparations. ^4^ Because of anticipated stability problems with liquid media, like degradation and microbiological contamination, this formulation is not appropriate for all pharmacologically active ingredients. ^4^ Patient-friendly dose forms such as Orodispersible tablets (ODT) were developed to overcome this. ^4^

ODT are “uncoated tablets that rapidly disintegrate in the oral cavity before being swallowed, turning into a solution or a suspension using the existing saliva and rarely requiring the use of external liquids such as water.” ^4^ Carbohydrates and polyols have been used in ODT formulations because they are highly soluble in water and have taste-improving properties. Polysaccharides and/or their corresponding alcohols are thus included in several excipient combinations developed for production. The ODT formulations rely on sugars and sugar alcohols. ^4^

People of all ages are affected by tooth wear. Dietary erosion is one such factor of tooth wear which is evident in those who consume acidic fruit juices and carbonated soft beverages as well as in patients on specific acidic oral drugs. ^5^ Clinical studies have demonstrated the erosive detrimental effects of vitamin supplements in chewable and lozenge forms, ^6^ and respiratory medications. ^7^ Additionally, research conducted *in vitro* has demonstrated the demineralizing capability of iron syrups ^8^, effervescent nutritional preparations, ^9^ mouthwashes, ^10^ and drugs used to treat phenylketonuria ^11^ and kidney disease. ^12^ The findings of these studies raised concerns about the adverse dental effects of taking these medicines with a potential of hydrogen (pH) ≤ 5.5 (critical pH). ^2^^,^^13^

Hellwig and Lussi ^14^ highlighted that those patients who are taking chewable and other dispersible forms of medications need to be aware of their erosive potential, which can cause drug-induced xerostomia. Pharmaceuticals typically employ acids as buffering agents to preserve stability, compatibility, enhance flavour and facilitate dissolution when they come into contact with water. ^15^ To maximize effectiveness and patient acceptance of ODTs, the pH needs to be adjusted. Therefore, preparations with low pH are frequently required for dispersion for better patient compliance. ^16^

The European Pharmacopoeia stipulates that ODTs must disintegrate in less than three minutes in the water to remain hassle-free ^17^ In contrast, the “FDA defines ODTs as forms that disintegrate in under thirty seconds in an *in vitro* setting.” ( https://www.fda.gov/regulatory-information/search-fda-guidance-documents/orally-disintegrating-tablets)

Only a few high-quality publications have addressed the role of quantity and frequency of sugar intake as an important factor in caries progression. ^18^^–^^20^ The routine use of LOMs and its relation to caries have been linked in numerous studies. ^21^^–^^24^ It is known that most of the common paediatric LOMs can cause dental erosion since their pH is below 5.5. ^13^^,^^15^^,^^25^ But the literature on ODT's erosive potential and sugar content in the Indian pharmaceutical market is lacking. With this background, we aimed to compare the total sugar content (TSC), pH, solubility, and Titratable Acidity (TA) with duration of use of drugs and among different drug classes of various ODTs.

## Methods

We conducted an *in vitro* study to evaluate the TSC, pH, solubility, and TA of commonly prescribed ODTs.

The study protocol was approved by “Kasturba Hospital and Kasturba Medical College Institutional Ethics Committee” (IEC2 – 22/2022).

The study involved two main phases: identification of ODTs and laboratory analysis.

### Identification of ODTs

*Preliminary survey*

A preliminary survey was conducted in Udupi city among paediatricians (n=4), and associated pharmacies (n=5), and pharmacists (n=8), about their most prescribed ODT. The participants were contacted personally with prior appointment and briefed about the study followed by written informed consent. Participants were chosen by convenience sampling. Subsequently, they were asked to list generic or brand names that are commonly prescribed to get a comprehensive list of ODTs used. According to the interviews, 22 drugs belonging to five different classes were identified. Further information was gathered to identify different manufacturers, which yielded 62 most prescribed ODTs ( Table 1). ODTs were further classified as long-term (anticonvulsants, anti-Tuberculosis, antivirals, antihistamines) and short-term medications (antibiotics, analgesics, and antiemetics).

**Table 1.** Distribution of potential of hydrogen (pH), solubility, titratable acidity (TA), and total sugar content (TSC) of different generic formulations of orodispersible tablets.

Generic name	N	pH		% Solubility		Total sugar (mg/tablet)		Titrable acidity in g/l	
		Mean	SD	Mean	SD	Mean	SD	Mean	SD
Montelukast	2	6.15	.92	68.15	8.05	14.36	1.45	2.28	1.16
Levocetirizine	2	6.98	.25	79.97	2.38	31.79	30.46	2.22	.
Domperidone	2	5.40	.57	70.69	3.66	45.13	4.36	7.36	.91
Ondansetron	3	6.30	1.21	79.30	15.96	12.31	4.47	7.39	7.07
Lansoprazole	1	6.50	.	52.64	.	28.72	.	1.91	.
Ketorolac	1	6.70	.	67.15	.	8.21	.	1.11	.
Paracetamol	4	6.48	.83	75.53	15.02	32.82	21.92	2.81	.96
Mefenamic Acid	1	6.63	.	65.69	.	11.28	.	14.26	.
Piroxicam	2	7.22	.65	70.25	.71	62.05	10.88	2.85	1.98
Clonazepam	9	6.24	.73	71.66	13.49	24.96	22.28	5.61	4.67
Lamotrigine	4	6.13	.35	57.09	14.63	27.18	28.87	5.72	2.90
Risperidone	1	7.00	.	66.17	.	4.10	.	.	.
Cefadroxil	3	5.60	.10	62.15	14.87	14.70	8.29	3.02	1.03
Cefalexin	2	5.30	.71	72.95	10.82	10.77	3.62	3.43	1.16
Cefixime	4	4.60	.88	43.76	1.73	23.34	15.20	3.86	1.62
Cefpodoxime	2	6.75	.64	37.96	3.19	26.67	2.90	.75	.
Amoxicillin	5	5.74	.93	36.47	4.10	14.36	4.70	2.15	.44
Amoxicillin and clavulinic acid	3	5.67	.12	42.77	12.76	27.01	10.37	1.79	.68
Azithromycin	2	7.80	1.27	30.40	4.67	42.57	16.68	.	.
Fluconazole	3	6.40	.40	67.66	18.83	20.86	21.90	2.21	.78
Isoniazid	3	6.10	.26	47.30	2.20	49.57	30.71	3.27	.
Acyclovir	3	5.67	.21	40.81	10.80	11.97	4.62	2.18	1.04

### Laboratory analysis

Determination of TSC, pH, solubility, and TA was carried out among the above identified ODTs in the department of Biochemistry, Kasturba Medical College, Manipal.

*Preparation of sample solution from the ODTs* ^2^

Each full dose of ODT was added to 50 mL distilled water (DW) and waited for three minutes for dissolution from which a 2 ml solution was pipetted into a test tube and further diluted with 40 ml DW. The solution was freshly prepared for each analysis.

*Measurement of pH* ^26^

A calibrated digital pH meter (“Eutec pH 700, Eutech Instruments Pte Ltd, Thermo Fisher Scientific India Inc, India”) accurate to 0.1 was used to determine the pH. The pH of the sample solution was displayed when the electrode was inserted into the 10 ml sample solution. The values were recorded in triplicate with three different samples and the average value was recorded.

*Estimation of TA* ^27^

A 40 ml sample solution was pipetted into a 100 ml conical flask. A 50 ml solution of 0.1 N NaOH was taken in a burette. Four drops of phenolphthalein indicator were added to the sample and it was titrated manually against the NaOH solution until a pale pink colour was achieved. The titration was conducted three times using a different ODT. The TA value was determined by multiplying the total amount of 0.1 N NaOH solution needed to neutralize the sample solution by a correction factor of 0.0978. Percentage TA was calculated using the following formula ^27^:

$$\text{“Percentage}\;\mathrm{TA}=\left[\left[\mathrm{ml}\;\text{NaOH used}\right]\;\times \;\left[0.1\;N\;\text{NaOH}\right]\;\times \;\left[\text{milliequivalent factor}\right]/\text{volume of the sample}\;\times \;1000\right]\;\times \;\left[100\right]\;\text{grams}\;\text{of sample”}$$

*Estimation of TSC* ^28^

The estimation of TSC was done by the colorimetric phenol sulphuric acid method which detects both reducing and nonreducing sugars. Stock sugar solution was freshly made by adding 50 mg of glucose and 50 mg of fructose in 100 ml of saturated benzoic acid (1mg/1ml) (0.42 gm of benzoic acid in 100 ml water). Working standard was prepared by diluting 10ml of stock solution in 100 ml of DW (1 ml = 100 mcg). To prepare dilutions with different concentrations the appropriate volume was taken in the series of test tubes. S1 – S6 (for ex: 0.2 ml = 20 μg of sugar concentration). A calibration curve was made using a series of standards (S1-S6) with concentrations of 20–120 μg. In two test tubes, 0.5 mL of a sample solution was taken. DW was used to fill each tube to a capacity of 2 ml. As a procedural control, 2 ml of DW was utilized as a blank. Each test tube received 0.05 mL of 80% phenol after which 5 mL of concentrated sulfuric acid was dispensed immediately into the solution. The test tubes were covered with parafilm (Bemis Company Inc, Neenah, WI, USA) and a 30-minute incubation period allowed the solution to develop its colour at room temperature. The optical density (OD) was read at 480nm by placing the samples in an auto colorimeter (LT-114, Labtronics, Haryana, India).

“TSC (gm/dL)=[(OD of test – OD of blank)/(OD of standard – OD of blank)]×concentration of standard×Dilution Factor”

*Percentage solubility* ^29^

A single ODT was added to 50 mL of DW. A pre-weighted filter paper (Grey kalpi filter paper, Saran hand-made paper industry, UP, India) was folded into a cone shape and placed in a glass funnel. The solution was poured into the funnel, and the filtrate was collected in a conical flask. The filter paper was air-dried for 24 hours until no moisture remained and then weighed. The difference between the weight of the filter paper before and after filtration gave the weight of the insoluble precipitate based on the below formula ^29^

$$\text{“Percentage solubility}=\left[\left(\text{weight of the tablet}–\text{weight of the precipitate}\right)/\text{weight of tablet}\right]\;\times \;100”$$

### Statistical analysis

All the analysis was done using SPSS version 20 (IBM Corp. IBM SPSS Statistics for Windows, Armonk, NY) (RRID:SCR_002865). A p-value of ≤0.05 was considered statistically significant. The normality of the data was evaluated using the Shapiro Wilk test. Inter-group comparisons of continuous variables were done using analysis of variance (ANOVA) with post-hoc Tukey's test or Kruskal Wallis ANOVA with Post-hoc Dunn's test based on the distribution of data among various drug classes of ODTs. Similarly, independent sample t-test or Mann-Whitney U test was used to compare with duration of use of ODTs. Sugar content was compared using the Mann-Whitney U test between different pH categories. Correlation between pH and TA was evaluated using Spearman’s rho. Underlying data for this study can be accessed at Mendeley Data. ^30^

## Results

Out of the 62 ODTs, four belonged to Antihistamines; five belonged to Anti-emetics and proton pump inhibitors; eight belonged to analgesics; 14 belonged to Anticonvulsants and antipsychotics; and 30 belonged to Antimicrobials (further divided into antibiotics, anti-tubercular, and anti-viral drugs).

The distribution of pH, percentage solubility, sugar content, and titratable acidity as per the generic content is listed in Table 1. The mean pH value for all the samples of medicines was 6.11±0.88 ( Table 2). The lowest mean pH, with respect to drug class, was seen in Antimicrobials (5.84±0.6) and the highest mean pH was seen in Analgesics (6.71±0.68) ( Table 3). There was no significant difference seen in the mean pH among the different drug classes (p=0.082) and between short-term (6.04±1.02) and long-term (6.22±0.61) ODTs ( Table 4).

**Table 2.** Descriptive statistics of overall potential of Hydrogen (pH), solubility, percentage sugar content, total sugar content (TSC) as per dose form, and percentage titratable acidity (TA) among the Orodispersible tablets.

Variable	Mean±SD	Range
pH	6.11±0.88	3.9-8.7
Solubility	59.11±17.97	27.09-96.96
Percentage sugar content	13.27±16.07	0-68.38
TSC as per dose form	25.13±19.5	0-70.77
Percentage TA	0.39±0.32	0-1.52

**Table 3.** Comparison of potential of hydrogen (pH), solubility, titratable acidity (TA), and total sugar content (TSC) among various drug classes of Orodispersible tablets.

	Antihistamines ^1^	Anti-emetics/proton pump inhibitor ^2^	Analgesics ^3^	Anticonvulsants/antipsychotic ^4^	Antimicrobials ^5^	P-value	Post-hoc test
	Mean± SD	Mean± SD	Mean± SD	Mean± SD	Mean± SD		
pH	6.56±0.73	6.03±0.95	6.71±0.68	6.26±0.64	5.84±0.96	0.082	-
Solubility	74.06±8.37	71.99±14.57	71.93±10.67	67.11±14.38	47.4±15.24	<0.001	1,2,3,4 >5
Percentage sugar content	16.13±22	24.33±17.34	11.38±9.27	22.97±23.31	6.67±7.7	0.01	2>5
TSC (mg/tablet)	23.08±20.28	25.98±16.5	34.36±24.84	24.10±23.07	23.25±17.16	0.718	-
Percentage TA	0.23±0.08	0.65±0.44	0.42±0.46	0.57±0.39	0.27±0.12	0.123	-
TA in g/l	2.26±0.82	6.28±4.32	4.21±4.58	5.65±3.96	2.6±1.18	0.127	-

**Table 4.** Comparison of potential of hydrogen (pH), solubility, titratable acidity (TA), and total sugar content (TSC) between short and long-term ODTs.

Variable	Duration		P-value
	Long-term	Short-term	
	Mean± SD	Mean± SD	
pH	6.22±0.61	6.04±1.02	0.409
Solubility	62.5±16.26	56.98±18.87	0.241
Percentage sugar content	18.68±20.85	9.86±11.17	0.23
TSC (mg/tablet)	25.6±23.24	24.83±17.06	0.568
Percentage TA	0.44±0.35	0.36±0.31	0.424
TA in g/l	4.37±3.49	3.55±3.04	0.337

The mean percentage solubility for all the ODTs was 59.11±17.97 ( Table 2). The mean percentage solubility was significantly different among various drug classes (p<0.001). The *post hoc* test showed least percentage solubility in Antimicrobials than other drug classes. No other significant differences were seen ( Table 3).

The sugar content in the ODTs was reported in two ways. Initially, the TSC was calculated per tablet (as per the dose form), followed by the percentage of sugar present per 100 mg ODT. The total mean sugar content and percentage of sugar were 25.13±19.5 and 13.28%±16.07, respectively ( Table 2). The lowest percentage of sugar content was seen in Antimicrobials (6.67%±7.70), and the highest percentage of sugar content was seen in Antiemetics and proton pump inhibitors (24.33%±17.34). The mean percentage sugar content was significantly different among the five drug classes. The post-hoc test showed that Antiemetics and proton pump inhibitors (24.33%±17.34) had significantly higher mean percentage sugar content than Antimicrobials (6.67%±7.7). No other significant differences were seen. The lowest TSC was seen in antihistamines (23.08%±20.28), while the highest was in Analgesics (34.36%±24.86). ( Table 3) The mean TSC for various drug classes did not significantly differ from one another (p=0.718). Similarly, there were no significant differences in the mean percentage sugar content and TSC between long-term ODTs and short tern ODTs (p=0.23 and 0.568) respectively ( Table 4).

The overall mean percentage TA was 0.39% ( Table 2). No significant difference was seen in the mean percentage TA between the five drug classes (p=0.123). Similarly, mean percentage TA did not significantly differ between the long-term (0.44%±0.35) and short-term (0.36%±0.31) ODTs (p=0.424) ( Table 4).

The pH, solubility, percentage sugar content, and TA showed no significant difference with respect to duration of use of ODT (p=0.409, p=0.241, p=0.23, p=0.424). A moderate significant negative correlation was seen between the pH and percentage TA (rho=-0.342; p=0.015).

The distribution of various ODTs as per the drug classes and critical pH was listed in Table 5. One-quarter of the ODTs (26%) had a mean pH below ≤5.5. There was no significant difference in the mean TSC and percentage sugar content with respect to critical pH (p=0.224 and 0.368) respectively ( Table 6).

**Table 5.** Distribution of different drug classes according to critical potential of hydrogen (pH).

Drug Class	pH	
	≤5.50	>5.50
	N(%)	N(%)
Antihistamines	1(6.2)	3(6.5)
Anti-emetics and proton pump inhibitor	2(12.5)	4(8.7)
Analgesics	1(6.2)	7(15.2)
Anticonvulsants and antipsychotic	2(12.5)	12(26.1)
Antimicrobials	10(62.5)	20(43.5)

**Table 6.** Comparison of mean percentage sugar content and total sugar content with critical potential of hydrogen (pH).

Variable	pH		P-value
	≤5.5	>5.5	
	Mean± SD	Mean± SD	
Percentage sugar content	8.4±10.55	14.97±17.37	0.368
TSC as per dose form	18.72±14.46	27.36±20.64	0.224

## Discussion

We conducted a comprehensive *in vitro* analysis of pH, solubility, sugar content, and TA among the commonly prescribed ODTs in the Udupi district, Karnataka, India. The spectrum of medications evaluated was quite broad. However, most of them were antimicrobials, which are less likely to be prescribed frequently and for a more extended period of time because their primary therapeutic indication is for short-term usage to treat acute infections. ^31^ Many excipients are used in the formulation of ODTs to ensure a good taste profile because they have a longer oral residence than other dose forms. ^32^

The overall mean pH for the total sample of ODTs was found to be 6.11 ± 0.88, which was less than that reported by Maguire *et al.* and Arora *et al.* (8.09 and 7.49, respectively). ^33^^,^^34^ We found that the pH was lowest in antimicrobials which were similar to Maguire *et al.* and Arora *et al.* (4.29, 5.7). ^2^^,^^33^ However, antimicrobials in liquid oral medications showed higher pH (6.4 and 6.36). ^3^^,^^35^ Analgesics in our study showed the highest pH (6.71), which was more than that reported in LOMs. ^13^^,^^15^^,^^35^^,^^36^ Antiemetics had a similar (6.03) pH as compared to Maguire *et al.* and Arora *et al.* (6.28 and 6.04, respectively). ^33^^,^^34^ Antihistamines had a pH of 6.56, while Maguire *et al.*, Arora *et al.*, Siddiq *et al.*, and Cavalcanti *et al.* reported pH less than the critical value (4.29, 4.8, 5.5, 4.1 respectively). ^26^^,^^33^^–^^35^ There was no significant difference in the pH between the short-term and the long-term medication, similar to the previous study. ^35^

TA is indicative of the erosive potential of drugs. ^37^ The overall TA of ODTs was 0.39 g/l, more than that reported by Maguire *et al.* and Arora *et al.* (0.04 and 0.06 g/l, respectively). ^33^^,^^34^ Antiemetics and proton pump inhibitors showed the highest TA (0.65 g/l), which was higher than previous studies conducted by Maguire *et al.* and Arora *et al.* (0.13 and 0.24 g/l respectively). ^33^^,^^34^ No significant difference was seen in the TA between long-term and short-term medications, similar to the previous study on LOM (Siddiq *et al.*). ^35^ Previous *in vitro* research showed that an acidic drugs can decrease enamel hardness and increase the enamel roughness. ^38^^–^^41^ Antimicrobials were the least water soluble seen among the different classes of drugs tested. A direct comparison could not be made as no previous studies were done on the solubility of ODTs.

We found that 98.37% (61) of the evaluated ODTs presented with sugar similar to previous studies on LOM. ^35^^,^^42^ But few studies on LOMs, showed the presence of sugar in 40-70% of the formulations. ^13^^,^^43^^,^^44^ Out of the 62 ODTs, only one medication in the anticonvulsant category (Lamitor DT) was sugar-free. Antiemetics and proton pump inhibitors showed the highest percentage of sugar content (24.33%), and the mean across all the drug classes was below 25%, which was lower than previous studies on LOM. ^45^ Our study showed no significant difference in the amount of sugar content in long-term and short term ODTs which was like that reported by Siddiq *et al.* ^35^

The long-term use of ODTs can be a potential risk for the development of caries especially among children. ^22^^,^^23^ According to Passos *et al.* only 5% sucrose concentration is required to form the cariogenic biofilm. ^45^ The majority of the ODTs contain carbohydrates as an excipient which plays a vital role in forming a tablet. ^46^ Despite available artificial substitutes, like sodium cyclamate, sodium saccharin, aspartame, ^47^ and sorbitol, ^48^ carbohydrates are used widely since they are economical and renewable material and can form tasteless, odorless, and biodegradable tablets which can disintegrate rapidly. The role of excipients and processing of ODTs can have substantial influence on the pH and sugar content. So ODTs of a particular drug may have different levels of pH and sugar content based on the dosage forms and can vary with different manufacturers. Hence, the current findings cannot be generalised to other similar available ODTs.

When metabolized by cariogenic microorganisms, fermentable sugars can induce a rapid pH drop below critical pH. ^49^ With lower pH among the ODTs, there can be a prolonged acidic environment in the oral cavity. Due to these factors, there can be a potential risk of dental erosion, decreased enamel hardness, increased roughness of tooth surface leading to plaque accumulation, demineralization of enamel, and rapid initiation and progression of dental decay. ^38^^–^^41^^,^^50^^,^^51^ Continuous usage of ODTs over a long time coupled with other risk factors like systemic comorbidities can cause the early onset and rapid progression of dentinal hypersensitivity, pain, rampant caries, and gum diseases. These can lead to poor aesthetics, functional limitations, and poor Oral Health-related Quality of Life.

Buffering capacity and salivary flow rate can vary among individuals with a substantial influence of comorbidities, pre-existing caries, age, gender, hormonal levels, diet, and medications. Healthcare personnel should consider the above factors before recommending ODTs, especially for long-term usage.

Overall, we can conclude that one in four ODT formulations had a pH below 5.5 (critical pH). Only one ODT formulation did not have a sugar content. No difference was seen in the pH, sugar content, and TA between the long-term and short-term ODTs. There were no differences seen in the pH, sugar content, and TA among drug classes. Antiemetics and proton pump inhibitors had higher sugar content than antimicrobials.

The exact contribution of the acidic potential of ODT on the tooth is unclear, especially in the oral cavity. The buffering capacity of saliva is a complex phenomenon and has the role of multiple host factors, which can vary significantly among individuals. Citric acid-containing ODTs with high TA and low pH can stimulate the salivary flow, thereby increasing the saliva’s buffering capacity. ^49^ The effect of salivary buffering capacity and plaque pH due to ODTs needs to be explored in the clinical scenario.

The comprehensive list of ODTs was limited to this geographic area. Hence, formulations of ODTs can vary in different countries and guidelines. Regular and long-term use of ODTs with longer oral clearance can be detrimental to oral health. Reformulating the medications as sugar-free can reduce the risk of various dental conditions as compared to the sugar containing formulations. ^52^ Manufacturers should consider reformulating these medications whenever feasible to minimize the potential risk of oral conditions.

Healthcare professionals should be aware of the potential risks of oral conditions due to the extended use of ODTs. Hence prescription of these ODTs should be limited to those individuals where compliance for swallowing is poor. Health care personnel should advise comprehensive oral examination and treatment of existing oral conditions before initiating extended ODT therapy. Also, individuals should be advised to follow at-home oral hygiene measures like gargling with water immediately after taking ODTs, use of fluoridated toothpaste, and brushing twice daily with or without supervision. Frequent dental examinations to monitor the oral conditions and progression of the same should be recommended. Preventive measures like topical fluoride applications, preventive sealants, and remineralising agents can be recommended to minimize the above oral conditions. However, sugar-free ODTs may contain polyols which may have laxative effects over long-term usage, and certain polyols are not recommended in children. ^53^

## Data Availability

Mendeley Data: Evaluation of sugar content and erosive potential of commonly prescribed Orally Dispersible Tablets- An in vitro study, https://doi.org/10.17632/w5z857k3cw.2. ^30^ This project contains the following underlying data:
- Mendeley Data Dr Lahari.xlsx - Mendeley extended Data Dr Lahari 03-02-23.xlsx (interview responses) Data are available under the terms of the Creative Commons Attribution 4.0 International license (CC-BY 4.0).
