# Supplementary material for: Sugar content and erosive potential of commonly prescribed Orodispersible tablets- An in vitro study
Source: F1000Res. 2023 Apr 12;12:228. Originally published 2023 Mar 1. [Version 2] doi: 10.12688/f1000research.130786.2 (PMC10114003; doi:10.12688/f1000research.130786.2)
Supplement: Peer review history

# Peer review: Reviewer response for version 1

**DOI:** 10.5256/f1000research.143566.r165216

**Published:** 2023-04-04

**Authors:** Harsh Priya

**Affiliations:** 1 Division of Public Health Dentistry, Centre for Dental Education and Research, All India Institute of Medical Sciences, New Delhi, Delhi, India

The article is innovative in nature. Congratulations to the authors.

 A few clarifications.

1. The mean TSC should not be expressed as percentage: “The total mean sugar content and percentage of sugar were 25.13%±19.5 and 13.28%±16.07, respectively ( Table 2)”
2. The figure entered needs to be rechecked for antimicrobials: “the post-hoc test showed that Antiemetics and proton pump inhibitors (24.33%±17.34) had significantly higher mean percentage sugar content than Antimicrobials (23.25%±17.16).”
3. The pH number can be mentioned. This would help in better understanding the comparability: “However, antimicrobials in liquid oral medications showed higher pH.”
4. English correction is suggested for the following sentence, “Buffering capacity and salivary flow rate can vary among individuals with the substantial influence of comorbidities, pre-existing caries, age, gender, hormonal levels, diet, and medications.”
5. A mention of confounding factors with suitable explanation is suggested.
6. Excipients can be further explained in the manuscript. The following reference can be helpful: Hannan *et al., *(2016) ^1^.
7. Newer innovations are formulated for caries prevention due to ODTs ^2^. 

Thank you.

 Best wishes

Is the work clearly and accurately presented and does it cite the current literature?

Yes

If applicable, is the statistical analysis and its interpretation appropriate?

Yes

Are all the source data underlying the results available to ensure full reproducibility?

Yes

Is the study design appropriate and is the work technically sound?

Yes

Are the conclusions drawn adequately supported by the results?

Yes

Are sufficient details of methods and analysis provided to allow replication by others?

Yes

Reviewer Expertise:

Public health Dentisry

I confirm that I have read this submission and believe that I have an appropriate level of expertise to confirm that it is of an acceptable scientific standard.

## References

[1] : Oral Dispersible System: A New Approach in Drug Delivery System. *Indian J Pharm Sci* .2016;78(1) : 10.4103/0250-474x.180244 2-7 10.4103/0250-474x.180244 27168675PMC4852571

[2] : Development of muco-adhesive orally disintegrating tablets containing tamarind gum-coated tea powders for oral care. *Int J Pharm X* .2019;1: 10.1016/j.ijpx.2019.100012 100012 10.1016/j.ijpx.2019.100012 31517277PMC6733284

# Peer review: Reviewer response for version 1

**DOI:** 10.5256/f1000research.143566.r165215

**Published:** 2023-03-27

**Authors:** Tarakant Bhagat

**Affiliations:** 1 Department of Public Health Dentistry, College of Dental Surgery, B.P. Koirala Institute of Health Sciences, Dharan, Nepal

This study compared the sugar content and erosive potential of commonly used orodispersible tablets (ODTs) in Udupi city of south India. Although the list of drugs was obtained from a single site, these are the most commonly used drugs throughout India. It was observed that one in four ODT formulations had a pH below 5.5 (critical pH); hence, regular and long-term use of ODTs with longer oral clearance can be detrimental to oral health. Recommendations like following up of at-home oral hygiene measures such as gargling with water immediately after taking ODTs, use of fluoridated toothpaste, and brushing twice daily with or without supervision, frequent dental examinations to monitor the oral conditions, preventive measures like topical fluoride applications, preventive sealants, and re-mineralising agents to minimize the oral conditions are important. 

 The article is well written.  This research will certainly be helpful to all the practitioners while prescribing such drugs, especially to children.

 In the results section, it is mentioned that a moderate significant negative correlation was seen between the pH and percentage TA, but the test used is not mentioned in the methods section. Please mention the name of the correlation test used in the relevant section.

Is the work clearly and accurately presented and does it cite the current literature?

Yes

If applicable, is the statistical analysis and its interpretation appropriate?

Yes

Are all the source data underlying the results available to ensure full reproducibility?

Yes

Is the study design appropriate and is the work technically sound?

Yes

Are the conclusions drawn adequately supported by the results?

Yes

Are sufficient details of methods and analysis provided to allow replication by others?

Yes

Reviewer Expertise:

Oral health, dental caries, oral cancer, oral health related quality of life, musculoskelatal disorders, burnout

I confirm that I have read this submission and believe that I have an appropriate level of expertise to confirm that it is of an acceptable scientific standard.
