# Supplementary material for: Sugar content and erosive potential of commonly prescribed Orodispersible tablets- An in vitro study
Source: F1000Res. 2023 Mar 1;12:228. [Version 1] doi: 10.12688/f1000research.130786.1 (PMC10114003; doi:10.12688/f1000research.130786.1)
Supplement: Peer review history

# Response

**Published:** 2023-04-13

**Authors:** Kalyana Pentapati

**Affiliations:** Manipal Academy of Higher Education, India

Query: The mean TSC should not be expressed as percentage: “The total mean sugar content and percentage of sugar were 25.13%±19.5 and 13.28%±16.07, respectively ( Table 2)”

 Response: Thank you. We have incorporated the change in the manuscript.

 Query: The figure entered needs to be rechecked for antimicrobials: “the post-hoc test showed that Antiemetics and proton pump inhibitors (24.33%±17.34) had significantly higher mean percentage sugar content than Antimicrobials (23.25%±17.16).”

 Response: Thank you for highlighting the typo. We have incorporated the change in the manuscript. “The post-hoc test showed that Antiemetics and proton pump inhibitors (24.33%±17.34) had significantly higher mean percentage sugar content than Antimicrobials (6.67%±7.7).”

 Query: The pH number can be mentioned. This would help in better understanding the comparability: “However, antimicrobials in liquid oral medications showed higher pH.”

 Response: Yes. Thank you. We have mentioned the same in the manuscript. (6.4 and 6.36)

 Query: English correction is suggested for the following sentence, “Buffering capacity and salivary flow rate can vary among individuals with the substantial influence of comorbidities, pre-existing caries, age, gender, hormonal levels, diet, and medications.”

 Response: We have revised the text in the manuscript. “Buffering capacity and salivary flow rate can vary among individuals with a substantial influence of comorbidities, pre-existing caries, age, gender, hormonal levels, diet, and medications.”

 Query: A mention of confounding factors with suitable explanation is suggested.

 Response: We have added this information. “The role of excipients and processing of ODTs can have substantial influence on the pH and sugar content. So ODTs of a particular drug may have different levels of pH and sugar content based on the dosage forms and can vary with different manufacturers. Hence, the current findings cannot be generalised to other similar available ODTs.”

 Query: Excipients can be further explained in the manuscript. The following reference can be helpful: Hannan *et al., *(2016) ^1^.

 Response: We have added this information. “Excipients in ODTs have a superdisintegrant, a bulking agent, a lubricant and swelling agent, a permeabilizing agent, sweeteners and flavorings agents which may have potential influence on the pH and sugar content of ODTs.”

 Query: Newer innovations are formulated for caries prevention due to ODTs ^2^. 

**References**

 1. Hannan PA, Khan JA, Khan A, Safiullah S: Oral Dispersible System: A New Approach in Drug Delivery System. *Indian J Pharm Sci*. 2016; **78** (1): 2-7 PubMed Abstract | Publisher Full Text

 2. Kiniwa R, Miyake M, Kimura SI, Itai S, et al.: Development of muco-adhesive orally disintegrating tablets containing tamarind gum-coated tea powders for oral care. *Int J Pharm X*. 2019; **1**: 100012

 Response: Thank you for your insight. These innovative products are yet to be proven for their effectiveness for caries prevention.

# Response

**Published:** 2023-04-13

**Authors:** Kalyana Pentapati

**Affiliations:** Manipal Academy of Higher Education, India

Query: In the results section, it is mentioned that a moderate significant negative correlation was seen between the pH and percentage TA, but the test used is not mentioned in the methods section. Please mention the name of the correlation test used in the relevant section.

 Response: We have added the information in the statistical analysis section of the manuscript. “Correlation between pH and TA was evaluated using Spearman’s rho.”
